# The tele-transition of toxicity management in routine oncology care during the severe acute respiratory syndrome (SARS-CoV-2) pandemic

**DOI:** 10.1038/s41416-020-01235-3

**Published:** 2021-02-09

**Authors:** Marika Rasschaert, Pieterjan Vanclooster, Tim Mertens, Ella Roelant, Katrien Lesage, Hans Prenen, Anke Verlinden, Ilse van Brussel, Jo Ravelingien, Annelies Janssens, Peter Van Dam, Marc Peeters

**Affiliations:** 1 Department of Oncology, Antwerp University Hospital Antwerp, Antwerp, Belgium; 2 Clinical Trials Center (CTC), CRC Antwerp, Antwerp University Hospital, University of Antwerp, Antwerp, Belgium; 3 Department of Information and Communication Technology, Antwerp University Hospital, Antwerp, Belgium; 4 Department of Haematology, Antwerp University Hospital, Antwerp, Belgium; 5 BVBA Remedus, Aartselaar, Belgium; 6 Department of Thoracic Oncology, Antwerp University Hospital, Antwerp, Belgium; 7 Unit of Gynecologic Oncology, Department of Obstetrics & Gynecology, Antwerp University Hospital, Antwerp, Belgium

**Keywords:** Cancer therapy, Health services, Signs and symptoms

## Abstract

**Background:**

Telehealth modalities were introduced during the SARS-CoV-2 pandemic to assure continuation of cancer care and maintain social distance.

**Methods:**

This is a retrospective cohort analysis of our telehealth expansion programme. We adapted two existing patient-reported outcome (PRO) telemonitoring tools that register and (self-)manage toxicities to therapy, while screening for SARS-CoV-2-related symptoms. Outpatients from a tertiary cancer centre were enrolled. The adapted PRO interface allowed for uniform registration of SARS-CoV-2-related symptoms and effective triage of patients at home where we also implemented systematic throat washings, when available.

**Results:**

Three hundred and sixty patients registered to the telemonitoring systems from March 13 to May 15, 2020. Four prespecified SARS-CoV-2 alarms resulted in three patients with positive PCR testing. Other Covid-19 symptoms (fever 5× and cough 2×) led to pretreatment triage resulting in 1 seroconversion after initial negative testing. One of the 477 throat washings proved positive.

**Conclusions:**

The rapid adoption of an amended PRO (self-)registrations and toxicity management system was feasible and coordinated screening for Covid-19. Continued clinical cancer care was maintained, with significant decreased waiting time. The systemic screening with throat washings offered no real improvement.

## Background

Digital health (also referred to as ehealth or telemedicine), in particular tele-consultations, have never been more relevant as during the coronavirus disease 2019 (Covid-19) pandemic.

The fight against the severe acute respiratory syndrome coronavirus 2 (SARS-CoV-2) infection highlighted some of the vulnerabilities in the health care systems around the world. As SARS-CoV-2 demanded more efforts in a limited and competing resource system, drastic changes were required to maintain the best care.

Oncologists around the world have been re-evaluating and individualising treatment plans for cancer patients to mitigate the exposure to and infection with the novel coronavirus,^1^ all the while balancing the potential benefit of containment measures with the negative health and social cost of postponing scheduled procedures. Cancer patients in particular are regarded as vulnerable to infections because of comorbidities and an immunosuppressive condition caused by the disease or the therapy.^2^ Recent publications indicated a higher incidence rate in infection and in development of severe events due to Covid-19.^3–5^ However, the effect of SARS-CoV-2 mortality in cancer patients is somewhat more co-dependent on underlying patient-specific factors or tumour type.^6–8^

One of the most striking (r)evolutions in times of SARS-CoV-2 is the accelerated implementation of telehealth in the transition from traditional in-person to web-based care models. Telehealth is not a novelty and stands for the provision of specialised care by a team of health care workers, doing so remotely and by means of a variety of telecommunication tools (through messaging, audio or video platforms). It can entail several facets in care provision: tele-consultation, tele-expertise, tele-surveillance, or tele-assistance and is well described.^9,10^ Telehealth has demonstrated high quality and acceptability by patients in the transition of care after a hospital discharge.^11^ More specifically in oncologic clinical care, tele-surveillance with use of patient-reported outcome (PRO) has proved to outperform clinical accuracy of toxicity scoring; it has also demonstrated validity, improved compliance to therapy and improved survival.^12–15^ PROs are defined by the US Food and Drug administration as any report made by the patient themselves about the status of a patient’s health condition without amendment or interpretation of the patient’s response by a clinician or anyone else.^16^ Thus telehealth, in all its facets, will enable the increased availability of different (molecular, biological, immunological and oral) therapies and careful management of symptoms or compliance thereof in the face of a rapidly changing oncological armamentarium.

**Fig. 1:**
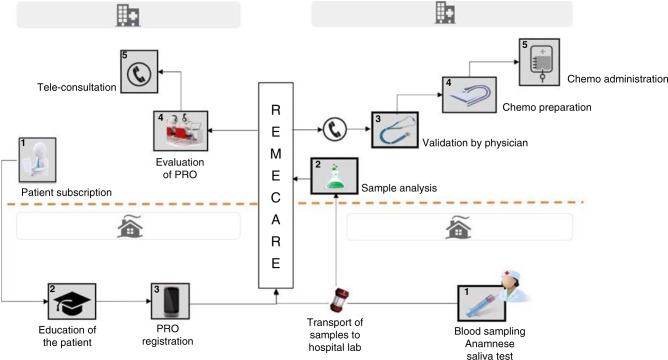
Transition of care. Illustration of two ambulatory monitoring systems, both registering data onto a central platform. Left: AMTRA (Ambulatory Monitoring of cancer TheRApy) and on the right side of the figure: BAPIC (Blood drawn in Ambulant Patients with Intravenous Cancer treatment). PRO patient-reported outcome.

We present a report on the rapid adoption of telehealth applications in an outpatient facility in a tertiary cancer centre in Belgium during the Covid-19 pandemic. Two patient-centred toxicity registration and (self-)management systems (“Blood drawn in Ambulant Patients with Intravenous Cancer treatment” and the “Ambulatory Monitoring of cancer Therapy”, respectively, BAPIC and AMTRA) were already installed to allow patients to (self-)register PROs onto a centralised RemeCare® platform (Fig. 1). Where the AMTRA system provides patients with the possibility to self-register (and manage) toxicities through a RemeCare® app, BAPIC will ensure intervention of the care team in case of severe toxicity scored by a trained nurse of a home care service, while blood is drawn at home. During the Covid-19 pandemic, broader implementation of these (amended) systems allowed for uniform SARS-CoV-2 symptom registry and safe triage of patients for further PCR screening.

We postulated that in the current landscape of a viral pandemic the RemeCare^®^ platform provides ways to allow care through (self-)management of toxicity and interaction with the care team, that it allows patient triage, a safe coordination of tests and continuous oncologic care.

## Materials and methods

### Development of AMTRA and BAPIC

Both the BAPIC and AMTRA systems are developed in collaboration with Remedus®, a home care service. The software was refined following previous experience with an in-house electronic tool that combined self-reporting of toxicity with self-management or care givers’ intervention.^17^ AMTRA is used by patients to self-register toxicities relative to their treatment. The symptom questionnaire builder in the RemeCare® app is manipulable to include such specificity both for patients to register and for the system’s algorithm to respond to the toxicity profile associated with intravenous or oral chemotherapy, immunotherapy and targeted treatments. (Supplemental Table 1). The clinical algorithm is developed to provide either self-management advice for toxicities graded as 1 or 2 or to alert a trained oncology nurse and the treating physician (care team) in case of severe (grade 3) or consecutive lower grade toxicity. The original protocol is described in a previous paper.^18^

During the present SARS-Cov-2 pandemic, this PRO registration and interaction system was amended to also screen for signs or symptoms of SARS-CoV-2 (COrona REmecare Oncology: COREO). For sure, SARS-CoV-2-related symptoms are routinely questioned by AMTRA’s question building/PRO system (fever, muscular pain, cough, shortness of breath). Thus, in case of fever (>38 °C) alone or coinciding with the registration of the three aforementioned symptoms in a single day (regardless of severity) an alarm would ensue in which case the patient would be contacted by the care team and screened at the hospital’s SARS-CoV-2 screening unit for formal PCR testing.

All data collected with the app were stored on the RemeCare® platform, which is password secured and linked to the Electronic Patient Report (EPR).

The technical components of the app and platform allowed secure transmission of data and storage in EPR. The app and the platform were attributed the CE label for medical devices class 2a by the Belgian Federal Agency for Medicines and Health Products in 2018 (https://www.famhp.be/en/human_use/health_products/medical_devices_accessories/generalities/ce_marking).

The BAPIC system provides transition of care by outsourcing certain clinical acts to the home care service, such as the screening for toxicities and the blood analysis prior to treatment.

The symptom questionnaire builder in the RemeCare® platform for BAPIC was not manipulable and the patients did not manage their own toxicities. A trained care giver from the care centre will verify and respond/interact to toxicities that are either serious (grade 3) or progressive (in regard to prior registrations). If the toxicity registration and the blood results allow it, the patient will receive a message to further organise the in-house scheduled treatment.

During the SARS-CoV-2 pandemic, this system allowed the trained nurse to screen for signs or symptoms of SARS-CoV-2.

### Saliva tests

From April 14 onward, we were able to offer our outpatients a routine testing through the use of throat washings. These tests were repeated every 2 weeks irrespective of symptoms and during the time active oncologic therapy was ongoing.

Throat washings were harvested by asking patients to oscillate over the posterior pharyngeal wall with 10 ml of sterile normal saline for 5–10 s, and then to spit out the saline from their throat to a sterile container. The throat washings were transferred to a biosafety 2 laboratory and were subjected to a reverse transcription (PCR) for SARS-CoV-2 (Covid-19) detection.^19,20^

### Patients

#### Usual care and SARS-CoV-2 risk-mitigating strategies

Usual care includes a consultation with an oncologist before the start of any systemic treatment to provide patients with verbal and written information on treatment benefits and expected toxicity; furthermore, instructions will be given on how to contact or attend the hospital for serious side effects.

Because of SARS-CoV-2, several mitigating risk strategies were implemented in routine cancer care from March 13 onward: non-urgent visits such as follow-ups were postponed or replaced by tele-consultation. All oncologic systemic treatments were continued and administered on an outpatient basis when possible. BAPIC was routinely offered to all patients to enable home blood sampling; Additional throat washing every fortnight was implemented when available from April 14 onward. Both BAPIC and AMTRA were used for systematic toxicity and SARS-CoV-2 symptom registration. Furthermore, masks for patients and health care providers were obligatory, visitation regulations were restrictive and social distancing was practiced as much as possible.

Patients enlisted in the different patient-centred PRO-registering systems from March 13 until May 15 were evaluated in the analysis. The patients were recruited in a tertiary cancer centre in Belgium, the University Hospital of Antwerp. All patients with malignant tumours receiving systemic anti-neoplastic agent(s) at any stage of their disease were recruited. Patients were required to provide informed consent, to be literate in Dutch or French and able to operate a smartphone.

Baseline demographic, tumour and treatment data were uploaded in a care request to the home care (nursing) organisation (Remedus®). Patients were contacted within several days by the home care nurse to organise a start-up visit at the patient’s home.

### Ethics

Patient registries and epidemiological data were captured in accordance to The General Data Protection Regulation (GDPR) Regulation (EU) 22016/679 (https://eur-lex.europa.eu/legal-content/EN/TXT/?uri=CELEX%3A02016R0679-20160504) on the protection of natural persons with regard to the processing of personal data and on the free movement of such data. The medical ethics committee of the Antwerp University Hospital has approved the outline of this cohort analysis (EC/PM/NVB/2020.075).

### Statistical analysis

Baseline patient characteristics were described using medians and ranges (minimum to maximum) for continuous variables. Qualitative variables were presented with observed numbers and percentages.

Any registration for a toxicity of grade ≥1 was considered as a positive registration.

The waiting time for a number of treatments before and during the Covid-19 pandemic were fitted in a linear mixed model with the period (before vs during) as a categorical fixed effect and the subject as a random effect. The same analysis was done with the number of a pre-specified chemotherapy regimen as outcome.

Statistical analysis was done using SAS 9.4.

## Results

### Patients

Three hundred and sixty patients were evaluated, 79 were recruited in the AMTRA system and 281 in the BAPIC system. Patients could have different tumour types or treatment incentives (e.g. adjuvant or metastatic) (Supplemental Table 2).

We lost 8 patients out of 79 in the AMTRA system, because they never started therapy at all (2 patients), because of progressive disease (1 patient) or because they did not recognise an added value of the tool (2 patients) or found it to be too cumbersome (3 patients). In the BAPIC system, 19 patients stopped early due to pre-planned stop of therapy or progressive disease (7 and 8 patients, respectively) and 4 patients dropped out because they preferred to have their blood taken in the hospital.

### Patient symptom burden

A total of 32,526 registrations were analysed; of which 15,374 were positive registrations. Some bothersome grade 3 toxicities are frequently scored by a minority of patients e.g. alopecia was scored 26 times by 4 patients; and the frequency of the registered grade 3 toxicity does not always reflect the earnestness of the side effects. We found that the most frequently grade 3 PROs are alopecia, anorexia and oral mucositis (Table 1). Furthermore, only nine instances of registered serious toxicity led to a clinical intervention.

**Table 1:** (Self-)registered grade 3 toxicity both in total number and per patient.

	AMTRA (*n* = 79) (registrations/patients)		BAPIC® (*n* = 86) (registrations/patients)	
Alopecia	26	4	NA	NA
Hand–foot syndrome	3	1	0	0
Dyspnoea	2	2	0	0
Nausea	1	1	10	6
Vomiting	0	0	2	2
Mucositis	6	4	7	4
Anorexia	21	9	0	0
Diarrhoea	1	1	0	0
Thirst	1	1	NA	NA
Insomnia	5	4	NA	NA
Anxiety	1	1	NA	NA
Pain	4	4	67	42
Covid alarm	5	5	0	0

*AMTRA* Ambulatory Monitoring of cancer TheRApy (a telemonitoring system with self-registration and management option), *BAPIC* Blood drawn in Ambulant Patient with Intravenous Cancer treatment (a telemonitoring system without self-management), *NA* not applicable.

### Intervention after SARS-CoV-2 alarms or related symptoms

We evaluated SARS-CoV-2-related symptoms separately and as a composite SARS-CoV-2 alarm, which was generated when patients had fever either alone or with the aforementioned symptoms: muscular pain, cough, shortness of breath, and in case of a combination of symptoms on the same day (without fever). Eleven patients were referred for testing after SARS-CoV-2 suspicion. Four SARS-CoV-2 alarms were generated, which ultimately were PCR-confirmed in 3 patients. One was hospitalised and a second patient quarantined at home. The third patient was a known SARS-CoV-2-infected man with oesophageal cancer who was tested and retested before he ultimately received chemotherapy in an isolated room because of protracted infection and consistent positive PCR testing.

Of those patients whom presented with fever as the only symptom (5 patients), only one patient demonstrated infection with the SARS-CoV-2. This patient initially tested negative but ultimately demonstrated a test conversion after he was hospitalised. One patient with both fever and cough (on separate occasions) was screened by his general practitioner and never tested for the virus. A final patient was diagnosed through nasopharyngeal swab, after which saliva testing proved positive (Table 2).

**Table 2:** SARS-Cov-2 patients: 11 patients with Covid-related symptoms and/or fever.

Age	Sex	WHO	Tumour type	Metastasis	Treatment	Alarm RemeCare/BAPIC		Test Covid-19	Result	Hospitalisation	Follow-up
						Date	Symptom				
66	M	2	Urothelial cancer	Liver/peritoneal	Carboplatin/Paclitaxel	20/03/2020	T° 39.2				Initially considered as tumour fever
						22/03/2020	T° 38.2				
						23/03/2020	T° 39.2	24/03/2020	Negative		Still hospitalised at present
						5/04/2020	T° 38.7				
								15/04/2020	Positive	15/04/2020	
74	M	1	Glioblastoma	None	Regorafenib	22/03/2020	Covid alarm	Netherlands	Positive	23/03/2020	Home isolation
						1/04/2020	Fever	16/04/2020			
54	M	0	Nasopharyngeal carcinoma	Bone/lung/pleura	Cisplatine/Gemcitabine	6/04/2020	Covid alarm	6/04/2020	Positive	6/04/2020	Admitted to hospital, discharged 24/3/2020
						7/04/2020	Fever	21/04/2020	Positive		
62	M	1	Urothelial cancer	Lung	Carboplatin/Paclitaxel	7/04/2020	Dyspnoea/cough/myalgie	Not tested			
						20/04/2020	T° 38				Pharyngitis according to GP
											No symptoms at present
54	M	0	Rectal adenocarcinoma	None	CAPOX	19/04/2020	T° 38.2	5/04/2020	Negative		No retesting No symptoms
61	M	1	Oesophageal	Bone	Cisplatin/5FU	18/05/2020	Covid alarm	30/03/2020 20/04/2020 05/05/2020 25/05/2020	Negative Positive Positive Negative	NA	Home isolation Received treatment in isolated room from 07/05/2020
68	M	2	Prostate	Bone	Taxotere	17/04/2020 18/04/2020 19/04/2020	Fever	20/04/2020	Negative	22/04–10/05	PD Stop therapy
45	M	2	Pancreatic	None	Folfirinox	25/05/2020	Covid alarm	15/04/2020 21/04/2020 24/04/2020 16/06/2020	Negative Negative Negative Negative	14/04–4/05 NA	PD New liver mets BSC Phone call
56	F	2	Ovarian	Liver/peritoneal	Caelyx	06/04/2020	Fever	16/04/2020	Negative	15/04/2020–27/04/2020	Death
64	F	1	Pancreatic	Liver/peritoneal	Gemcitabine/Abraxane	20/04/2020	Fever	11/05/2020 26/05/2020	Negative Negative	NA	Tumour fever
68	M	1	Lung	Lung/suradrenal gland	Pembrolizumab (stop due to colitis not related)	15/04/2020	Cough Dyspnoe	15/04/2020 13/05/2020	Positive Positive	NA	Home isolation

*NA* not applicable, *PD* new liver.

### Chemotherapy administration at the outpatient facility

To evaluate the effect of coordinated screening on the outpatient facility, we compared three specific treatment regimens given during the lock down period in the SARS-CoV-2 pandemic of 2020 and the same period of time in 2019. These treatment types were considered routine and could be administered for different indications.

We found no significant difference in number of treatments during the SARS-CoV-2 lock down period and the similar period of 2019, but a significant decline in waiting time for both the Folfox/Folfiri schedule and Paclitaxel regimen (*p* < 0.0001). In case of Nivolumab administrations, we evaluated a shortened waiting time (*p* < 0.0001) and a diminished number of applications (*p* = 0.007). However, this was to be expected since we opted to administer Nivolumab routinely in a 4-week schedule, whereas in 2019 we preferred to start a nivolumab treatment schedule with a bi-weekly administration (Table 3).

**Table 3:** Number of chemotherapy administrations and waiting time before administration of drugs: during the SARS-Cov-2-related pandemic and a similar period in 2019.

	Waiting time (min)			Number of treatments		
	Pre SARS-Cov-2	SARS-Cov-2	*p* and CI for diff	Pre SARS-Cov-2	SARS-Cov-2	*p* and CI for diff
Folfox/Folfiri	123.4 (5.1)	87.1 (6.6)	<0.0001 −36.3 [−52.1, −20.6]	3.15 (0.22)	3.21 (0.28)	0.848 0.07 [−0.64, 0.78]
Paclitaxel	133.2 (7.3)	85.2 (6.4)	<0.0001 −48.0 [−67.3, −28.6]	4.25 (0.75)	3.88 (0.65)	0.709 −0.38 [−2.42, 1.67]
Nivolumab	102.6 (5.7)	56.0 (7.0)	<0.0001 −46.6 [−62.9, −30.3]	3.53 (0.23)	2.52 (0.28)	0.007 1.02 [0.29, 1.74]

Reported values are estimated mean (SE), *p* value period and 95% CI for difference Covid-19 min pre-Covid-19 from the linear mixed model.

### Saliva test

When available, we offered a systematic screening tests through throat washings, every fortnight. This procedure was performed at home, synchronous with a pretreatment blood sample taken by the trained home care nurses. From April 14 until May 15, we evaluated 477 throat washings to find 1 positive (this patient was already known to be infected).

## Discussion

The Covid-19 pandemic spread around the world in a mere few weeks outpacing each and every health care system’s ability to track, test and confine infected patients. Physical distancing has become the primary control method to limit the impact of Covid-19, and so it will remain while we await better care and cure options. Consequently, oncologists were forced to rethink the delivery of cancer care. It was obvious that a centralised model of in-person and in-hospital interactions between vulnerable patients or between patients and their clinicians could potentially lead to the spread of the virus. Faced with this difficult choice, many physicians and care systems needed to postpone scheduled care, potentially leading to negative health and social cost (in case of postponing currative intent interventions such as surgical procedures and (neo-) adjuvant therapies).

In this retrospective cohort analysis, we reported on the rapid adoption of a PRO registration and toxicity management app and platform (Supplemental Fig. 1).

Furthermore, through minor adaptations it provided an efficient screening and triage tool, coordinating appropriate care for a particular vulnerable patient group, while maintaining scheduled therapy appointments.

In evaluating the alarms generated, the composite SARS-CoV-2 alarm performed better in coordinating screening efforts than either SARS-CoV-2-related symptom separately. However, the analysis is retrospective in nature and on a small cohort of patients and we did not evaluate for anosmia, which is recognised as a Covid-19-related symptom. Second, the difference in toxicity scoring when performed by patients or through the help of home care nurse (AMTRA system vs BAPIC system), as demonstrated in Table 1, illustrates the paradigm that PROs outperform clinician’s evaluation of toxicity.

Through the use of coordinated screening, we were able to continue oncologic care as we demonstrated for specific schedules; what’s more, we improved on efficacy with shorter waiting times.

During this viral pandemic, we radically and rapidly abandoned our centralised face-to-face model of care in order to prioritise accurate care for our patients and to avoid the spread of the virus to uninfected patients seeking evaluation or treatment. Some regulatory changes have been made in response to Covid-19 in order to support an accelerated expansion of telehealth. However, many challenges remain such as the expansion of the reimbursement regulations, capacity and access to care, privacy issues and safety regulations.

In Belgium, we have seen the swift adoption of reimbursement for telephone consultation. Still, telehealth comprises more extensive interaction options awaiting formal payment regulation. Although previous data suggest that clinicians are broadly supportive of telemedicine^21^, low reimbursement will be regarded as a critical disincentive, because, any system, where a large amount of the electronic data screening and symptom triage is done through trained health care workers, will reallocate nurses and physicians from the hospital duties to the computer desk.

Regulatory changes governing payment parity will need to be addressed after the pandemic because adequate reimbursement for telehealth will be an important factor to maintaining broad adoption.^22^ It is evident that patients will not benefit from telehealth if physicians are not incentivised to propose the option beyond the scope of a pandemic. However, if telehealth would be a more economical way to deliver health care, this may represent a financial threat to smaller practices and centres,^21^ necessitating patients to travel further for specialised care treatments.

Beyond the reimbursement issues, other hurdles remain; language, age and electronic aptitude will have to be addressed, to minimise drop out and failed inclusions. Across all phases of the cancer treatment, continuum extra access to support will have to be organised. Remote interpreter services, when needed, and responsive technical help for those patients who are less accustomed with ehealth are just a few of the options. Furthermore, the option for telephone visits should be to ensure that all patients receive virtual care regardless of technology access or knowledge.^23^

Finally, while in the response to SARS-CoV-2 privacy may not have been the most important concern; in moving forward, due diligence is called for to ensure security and safety of data under the European rule of 2016 (GDPR).

On 19 March 2020, the European Data Protection Board adopted a formal statement on the processing of personal data in the context of the SARS-CoV-2 outbreak. It emphasised that *the data protection regulation, such as GDRP*, is a broad legislation and provides for the rules to apply to the processing of personal data in a context such as the one relating to Covid-19. Indeed, the GDPR provides for the legal grounds to enable the employers and the competent public health authorities to process personal data in the context of epidemics, without the need to obtain the consent of the data subject. The processing of personal data should be done for specific and explicit purposes and the data subject should have received transparent information about these processing activities (https://edpb.europa.eu/news/news/2020/statement-edpb-chair-processing-personal-data-context-covid-19-outbreak_nl). All in all, currently it seems to be indeed allowed to use technologies, tools or practices deviating from usual data security standards in medical devices, provided that this is absolutely necessary in the fight against the Covid-19 outbreak, the security measures envisaged ensure an adequate level of security and a right balance is struck between the right to personal data protection and the rights to health care, medical treatment and health security, as this is to be evidenced by a dedicated ad hoc assessment.

As the use of these digital tools will become more mainstream in cancer care, sensitive information will have to be protected. Both patients and practitioners must be informed about the risks and best practices while on a digital platforms. It is essential that these platforms will ensure safety and privacy regardless of which EPR internet provider of telehealth tool used.^22^

In conclusion, this paper describes the experiences with an adapted version of an existing, customisable system for home-based symptom and side effect reporting in order to allow for efficient triage prior to patient-centred clinical workflow during the SARS-CoV-2 pandemic, reassuring continuation of care.

The Current surge in use of telehealth needs to evolve into a more sustainable long-term model.

## Supplementary information

Dataset 1, Table 2, Figure 1

## Data Availability

The data from different ehealth tools are available via a secure (password protected) web-based platform. Due to privacy issues, this platform cannot be made public. However, the selected and anonymised records for the patients treated in the University Hospital of Antwerp are available on file with Remedus®.
