# Supplementary material for: The tele-transition of toxicity management in routine oncology care during the severe acute respiratory syndrome (SARS-CoV-2) pandemic
Source: Br J Cancer. 2021 Feb 9;124(8):1366–72. doi: 10.1038/s41416-020-01235-3 (PMC8039036; doi:10.1038/s41416-020-01235-3)
Supplement: Supplementary file 1 — Dataset 1, Table 2, Figure 1 [file 41416_2020_1235_MOESM1_ESM.docx]

Supplemental data

Supplemental table 1: Adaptation of the NCI’s PRO CTCAE : shortened and translated in French and Dutch with its reciprocal and automated responses or suggestions

### Braken/ Vomissements

|  | **Graden** | **Verstrek info** |
| --- | --- | --- |
| Gr0 | Afwezig/ Absent |  |
| Gr1 | Ik braak niet meer dan 2 keer per dag/ Je ne vomis pas plus de deux fois par jour | Zorg voor voldoende vochtinname: drink 1,5 tot 2 liter vocht per dag (water, cola (evt. laten uitbruisen), appelsap, limonade, isotone sportdranken, thee of bouillon). Dwing uzelf niet om te eten, hebt u toch zin om te eten, neem dan een hartig of zoet tussendoortje, dit levert veel calorieën in een klein volume. Neem kleinere porties, maar eet meerdere keren per dag./ Veillez à vous hydrater suffisamment : buvez 1,5 à 2 litres par jour (eau, coca-cola dont on élimine éventuellement les bulles, jus de pommes, limonade, boissons énergisantes isotones, thé ou bouillon). Forcez-vous à ne pas manger. Si vous avez quand même faim, nourrissez-vous d’un en-cas copieux ou sucré, source d’un nombre élevé de calories par petit volume. Consommez de plus petites portions, plusieurs fois par jour. |
| Gr2 | Ik braak 3 tot 5 keer per dag/ Je vomis de trois à cinq fois par jour | Neem medicatie tegen braken (volgens advies van uw arts). Neem contact op met uw huisarts of het ziekenhuis indien het niet overgaat. Zorg voor voldoende vochtinname: drink 1,5 tot 2 liter vocht per dag (water, cola (evt. laten uitbruisen), appelsap, limonade, isotone sportdranken, thee of bouillon). Dwing uzelf niet om te eten, hebt u toch zin om te eten, neem dan een hartig of zoet tussendoortje, dit levert veel calorieën in een klein volume. Neem kleinere porties, maar eet meerdere keren per dag./ Prenez des médicaments contre les vomissements (selon l’avis de votre médecin). Contactez votre médecin traitant ou l’hôpital si cela ne passe pas. Veillez à vous hydrater suffisamment : buvez 1,5 à 2 litres par jour (eau, coca-cola dont on élimine éventuellement les bulles, jus de pommes, limonade, boissons énergisantes isotones, thé ou bouillon). Forcez-vous à ne pas manger. Si vous avez quand même faim, nourrissez-vous d’un en-cas copieux ou sucré, source d’un nombre élevé de calories par petit volume. Consommez de plus petites portions, plusieurs fois par jour. |
| Gr3 | Ik braak tot 6 keer of meer per dag/ Je vomis jusqu’à six foisou plus par jour | Uw registratie is nu beschikbaar voor uw zorgteam. Indien u ongerust bent over dit symptoom, gelieve dan het centrum van uw behandelende arts te contacteren of bezoek de dienst spoedgevallen./ Votre enregistrement est maintenant disponible pour votre équipe de soins. Si ce symptôme vous inquiète, veuillez contacter le centre de votre médecin traitant ou vous rendre aux urgences. |

### Misselijkheid/ Nausée

|  | **Graden** | **Verstrek info** |
| --- | --- | --- |
| Gr0 | Geen klachten/ Aucune plainte |  |
| Gr1 | Lichte misselijkheid die continu aanwezig is, onafhankelijk van het moment van voeding/ Légère nausée permanente indépendante du moment de la nutrition | Zorg voor voldoende vochtinname: drink 1,5 tot 2 liter vocht per dag (water, cola (evt. laten uitbruisen), appelsap, limonade, isotone sportdranken, thee of bouillon). Dwing uzelf niet om te eten, hebt u toch zin om te eten, neem dan een hartig of zoet tussendoortje, dit levert veel calorieën in een klein volume. Neem kleinere porties, maar eet meerdere keren per dag./ Veillez à vous hydrater suffisamment : buvez 1,5 à 2 litres par jour (eau, coca-cola dont on élimine éventuellement les bulles, jus de pommes, limonade, boissons énergisantes isotones, thé ou bouillon). Forcez-vous à ne pas manger. Si vous avez quand même faim, nourrissez-vous d’un en-cas copieux ou sucré, source d’un nombre élevé de calories par petit volume. Consommez de plus petites portions, plusieurs fois par jour. |
| Gr2 | Ernstige misselijkheid die continu aanwezig is, onafhankelijk van het moment van voeding/ Forte nausée permanente indépendante du moment de la nutrition | Neem medicatie tegen misselijkheid (volgens advies van uw arts). Neem contact op met uw huisarts of het ziekenhuis indien het niet overgaat. Zorg voor voldoende vochtinname: drink 1,5 tot 2 liter vocht per dag (water, cola (evt. laten uitbruisen), appelsap, limonade, isotone sportdranken, thee of bouillon). Dwing uzelf niet om te eten, hebt u toch zin om te eten, neem dan een hartig of zoet tussendoortje, dit levert veel calorieën in een klein volume. Neem kleinere porties, maar eet meerdere keren per dag./ Prenez des médicaments contre les nausées (selon l’avis de votre médecin). Contactez votre médecin traitant ou l’hôpital si cela ne passe pas. Veillez à vous hydrater suffisamment : buvez 1,5 à 2 litres par jour (eau, coca-cola dont on élimine éventuellement les bulles, jus de pommes, limonade, boissons énergisantes isotones, thé ou bouillon). Forcez-vous à ne pas manger. Si vous avez quand même faim, nourrissez-vous d’un en-cas copieux ou sucré, source d’un nombre élevé de calories par petit volume. Consommez de plus petites portions, plusieurs fois par jour. |
| Gr3 | Ernstige misselijkheid die toeneemt bij start voedselinname en weer afneemt bij stoppen van de voedselinname/ Nausée sévère qui augmente au début de la nutrition et diminue de nouveau après l’arrêt de celle-ci | Uw registratie is nu beschikbaar voor uw zorgteam. Indien u ongerust bent over dit symptoom, gelieve dan het centrum van uw behandelende arts te contacteren of bezoek de dienst spoedgevallen./ Votre enregistrement est maintenant disponible pour votre équipe de soins. Si ce symptôme vous inquiète, veuillez contacter le centre de votre médecin traitant ou vous rendre aux urgences. |

### Dorstgevoel en frequent plassen/ Sensation de soif et miction fréquente

Beg. tekst : Heeft u het gevoel meer dorst te hebben?/ Avez-vous l’impression d’avoir plus de soif ?

|  | **Graden** | **Verstrek info** |
| --- | --- | --- |
| Gr0 | Nee: ik moet niet frequenter plassen dan voorheen en heb geen verhoogd dorstgevoel/ Non : je ne dois pas uriner plus fréquemment qu’avant et ma sensation de soif n’a pas augmenté |  |
| Gr1 | Ja: ik moet frequenter plassen dan voorheen en heb een sporadisch dorstgevoel dat meteen overgaat bij toedienen van vocht/ Oui : je dois uriner plus fréquemment qu’avant et ma sensation de soif, sporadique, disparaît immédiatement quand je m’hydrate | Vermijd suikerhoudend eten en drank. / Éliminez les boissons et les aliments sucrés. |
| Gr2 | Ja: Ik moet frequenter plassen, en heb regelmatig dorstgevoel/ Oui: je dois uriner fréquemment, j’ai souvent une sensation de soif. | Vermijd suikerhoudend eten en drank. Vraag indien nodig verder advies bij uw oncologisch team./ Éliminez les boissons et les aliments sucrés. Si nécessaire, demandez conseil auprès de votre équipe oncologique. |
| Gr3 | Ja: ik moet frequenter plassen dan voorheen en dit in combinatie met een aanhoudend dorstgevoel en nachtelijk plassen/ Oui : je dois uriner plus fréquemment qu’avant et ma sensation de soif persiste. Je dois aussi uriner la nuit | Uw registratie is nu beschikbaar voor uw zorgteam. Indien u ongerust bent over dit symptoom, gelieve dan het centrum van uw behandelende arts te contacteren of bezoek de dienst spoedgevallen./ Votre enregistrement est maintenant disponible pour votre équipe de soins. Si ce symptôme vous inquiète, veuillez contacter le centre de votre médecin traitant ou vous rendre aux urgences. |

### Hand/voet huid reactie/ Main/pied reaction de peau

|  | **Graden** | **Verstrek info** | |  |
| --- | --- | --- | --- | --- |
| Gr0 | Geen klachten | |  | |
| Gr1 | Minimale huidveranderingen zonder pijn (bv. roodheid) | | - Neem geen hete douches, baden en ga niet in de sauna - Droog handen en voeten volledig, deppen i.p.v. wrijven - Stel uw huid niet bloot aan de zon, zoek frisse en schaduwrijke plaatsen - Draag geen spannende of afsluitende schoenen of sokken - Draag geen juwelen (ringen, armbanden) - Beperk hevige wrijving of kneuzing van de handen en de voeten: vermijd lange wandelingen, jogging, gebruik van gereedschap dat wrijving en druk veroorzaakt ter hoogte van uw handen (knutselen, tuinieren), zware huishoudelijke taken (bijvoorbeeld langdurig gebruik van de mixer) - Gebruik geen zelfklevende verbanden en pleisters - Gebruik vocht inbrengende lotions om kloven te voorkomen - Gebruik handschoenen bij het schoonmaken en werken in de tuin - Droog handen en voeten volledig, zonder te wrijven - Gebruik eventueel ijs ter hoogte van uw handen en voeten om de pijn te verlichten - Ne prenez pas de douches et de bains brûlants et n’allez pas au sauna - Séchez-vous complètement les mains et les pieds, tamponnez au lieu de frotter - N’exposez pas votre peau au soleil, recherchez des endroits frais et ombragés - Ne portez pas de chaussures ou de chaussettes serrées ou fermées - Ne portez pas de bijoux (bagues, bracelets) - Limitez les frottements intensifs ou les contusions aux mains et aux pieds : évitez les longues promenades, le jogging, l’utilisation d’outils qui provoquent des frottements et des pressions au niveau des mains (bricolage, jardinage), les tâches ménagères lourdes (par exemple l’utilisation prolongée du mixeur) - N’utilisez pas de pansements ou de sparadraps adhésifs - Utilisez des lotions hydratantes pour prévenir les fissures - Portez des gants pour le nettoyage et le jardinage - Séchez-vous complètement les mains et les pieds, sans frotter - Servez-vous éventuellement de glace pour vos mains et vos pieds afin d’atténuer la douleur | |
| Gr2 | Huidveranderingen met pijn (bv. blaren, kloven, bloeden), stoort de dagdagelijkse activiteiten (bv. koken, winkelen) | | - Neem geen hete douches, baden en ga niet in de sauna - Droog handen en voeten volledig, deppen i.p.v. wrijven - Stel uw huid niet bloot aan de zon, zoek frisse en schaduwrijke plaatsen - Draag geen spannende of afsluitende schoenen of sokken - Draag geen juwelen (ringen, armbanden) - Beperk hevige wrijving of kneuzing van de handen en de voeten: vermijd lange wandelingen, jogging, gebruik van gereedschap dat wrijving en druk veroorzaakt ter hoogte van uw handen (knutselen, tuinieren), zware huishoudelijke taken (bijvoorbeeld langdurig gebruik van de mixer) - Gebruik geen zelfklevende verbanden en pleisters - Gebruik vocht inbrengende lotions om kloven te voorkomen - Gebruik handschoenen bij het schoonmaken en werken in de tuin - Droog handen en voeten volledig, zonder te wrijven - Gebruik eventueel ijs ter hoogte van uw handen en voeten om de pijn te verlichten - Neem 1000 mg (1g) paracetamol (bvb. Dafalgan®), maximum 4x per dag - Ne prenez pas de douches et de bains brûlants et n’allez pas au sauna - Séchez-vous complètement les mains et les pieds, tamponnez au lieu de frotter - N’exposez pas votre peau au soleil, recherchez des endroits frais et ombragés - Ne portez pas de chaussures ou de chaussettes serrées ou fermées - Ne portez pas de bijoux (bagues, bracelets) - Limitez les frottements intensifs ou les contusions aux mains et aux pieds : évitez les longues promenades, le jogging, l’utilisation d’outils qui provoquent des frottements et des pressions au niveau des mains (bricolage, jardinage), les tâches ménagères lourdes (par exemple l’utilisation prolongée du mixeur) - N’utilisez pas de pansements ou de sparadraps adhésifs - Utilisez des lotions hydratantes pour prévenir les fissures - Portez des gants pour le nettoyage et le jardinage - Séchez-vous complètement les mains et les pieds, sans frotter - Servez-vous éventuellement de glace pour vos mains et vos pieds afin d’atténuer la douleur - Prenez 1 000 mg (1 g) de paracétamol (p.ex. Dafalgan®), maximum 4 x par jour | |
| Gr3 | Ernstige huidveranderingen met pijn (bv. blaren, kloven, bloeden), stoort de zelfzorg (bv. wassen, eten) | | Uw registratie is nu beschikbaar voor uw zorgteam. Indien u ongerust bent over dit symptoom, gelieve dan het centrum van uw behandelende arts te contacteren of bezoek de dienst spoedgevallen./ Votre enregistrement est maintenant disponible pour votre équipe de soins. Si ce symptôme vous inquiète, veuillez contacter le centre de votre médecin traitant ou vous rendre aux urgences. | |

### Diarree /Diarhée

|  | **Graden** | **Verstrek info** |
| --- | --- | --- |
| Gr0 | Ik heb niet meer ontlasting dan normaal/ Je ne vais pas plus à selles que la normale |  |
| Gr1 | Ik heb 1-3 keer meer ontlasting dan normaal, de consistentie is los/Je vais à selles 1 à 3 fois plus que la normale. La consistance de mes selles est molle | - Bij 1e losse stoelgang => 2 smelttabletten of 2 capsules loperamide (4 mg) - Bij 2e losse stoelgang => 1 smelttablet of 1 capsule loperamide (2 mg) - Bij 3e losse stoelgang => 1 smelttablet of 1 capsule loperamide (2 mg)   Voedingsadvies bij diarree:   - Zorg voor voldoende vochtinname: drink 1,5 tot 2 liter vocht per dag - Vermijd te koude en te warme dranken - Vermijd een te vette voeding - Vermijd een te sterk gekruide voeding - Vermijd vers fruit (sinaasappelen) en rauwkost - - Lors des 1res selles molles => 2 comprimés orodispersibles ou 2 capsules de lopéramide (4 mg) - - Lors des 2es selles molles => 1 comprimé orodispersibles ou 1 capsule de lopéramide (2 mg) - - Lors des 3es selles molles => 1 comprimé orodispersibles ou 1 capsule de lopéramide (2 mg) - Conseils nutritionnels en cas de diarrhée : - - Veillez à vous hydrater suffisamment : buvez 1,5 à 2 litres par jour - - Évitez les boissons trop froides et trop chaudes - - Ne mangez pas trop gras - - Ne mangez pas trop épicé - - Évitez les fruits frais (oranges) et les crudités |
| Gr2 | Ik heb 4-6 keer meer ontlasting dan normaal, de consistentie is los/ Je vais à selles 4 à 6 fois plus que la normale. La consistance de mes selles est molle | - Bij 4e losse stoelgang => 1 smelttablet of 1 capsule loperamide (2 mg) - Bij 5e losse stoelgang => 1 smelttablet of 1 capsule loperamide (2 mg) - Bij 6e losse stoelgang => 1 smelttablet of 1 capsule loperamide (2 mg)   Voedingsadvies bij diarree:   - Zorg voor voldoende vochtinname: drink 1,5 tot 2 liter vocht per dag - Vermijd te koude en te warme dranken - Vermijd een te vette voeding - Vermijd een te sterk gekruide voeding - Vermijd vers fruit (sinaasappelen) en rauwkost - - Lors des 4es selles molles => 1 comprimé orodispersibles ou 1 capsule de lopéramide (2 mg) - - Lors des 5es selles molles => 1 comprimé orodispersibles ou 1 capsule de lopéramide (2 mg) - - Lors des 6es selles molles => 1 comprimé orodispersibles ou 1 capsule de lopéramide (2 mg) - Conseils nutritionnels en cas de diarrhée : - - Veillez à vous hydrater suffisamment : buvez 1,5 à 2 litres par jour - - Évitez les boissons trop froides et trop chaudes - - Ne mangez pas trop gras - - Ne mangez pas trop épicé - - Évitez les fruits frais (oranges) et les crudités |
| Gr3 | Ik heb > 6 keer meer ontlasting dan normaal, de stoelgang is helemaal vloeibaar, zonder vaste stukjes OF de buikloop verstoort mijn dagelijks leven/ Je vais à selles plus de 6 fois que la normale. La consistance de mes selles est tout à fait liquide, sans morceaux solides OU la diarrhée perturbe ma vie quotidienne | Uw registratie is nu beschikbaar voor uw zorgteam. Indien u ongerust bent over dit symptoom, gelieve dan het centrum van uw behandelende arts te contacteren of bezoek de dienst spoedgevallen.   - Bij 7e losse stoelgang => 1 smelttablet of 1 capsule loperamide (2 mg) - Soms kan diarree gepaard gaan met uitdrogingsverschijnselen. Die verschijnselen zijn een gevoel van dorst, een droge mond, een droge tong, een droge of gerimpelde huid en verminderde en donkere, troebele urine. - Votre enregistrement est maintenant disponible pour votre équipe de soins. Si ce symptôme vous inquiète, veuillez contacter le centre de votre médecin traitant ou vous rendre aux urgences.Lors des 7e selles molles => 1 comprimé orodispersibles ou 1 capsule de lopéramide (2 mg) - La diarrhée peut parfois s’accompagner de phénomènes de dessèchement. Ces derniers sont les suivants : sensation de soif, bouche et langue sèches, peau sèche et ridée, urine sombre et trouble en moindre quantité. |

### Diarree (IT)/ Diarhée (TI)

|  | **Graden** | **Verstrek info** |
| --- | --- | --- |
| Gr0 | Ik heb niet meer ontlasting dan normaal |  |
| Gr1 | Ik heb 1-3 keer meer ontlasting dan normaal, de consistentie is los | - Zorg voor voldoende vochtinname - Vermijd vezelrijke voeding - Vermijd melk en zuivelproducten - Hydratez-vous suffisamment - Évitez la nourriture riche en fibres - Évitez le lait et les produits laitiers |
| Gr2 | Ik heb 4-6 keer meer ontlasting dan normaal, de consistentie is los | - Zorg voor voldoende vochtinname - Vermijd vezelrijke voeding - Vermijd melk en zuivelproducten - Hydratez-vous suffisamment - Évitez la nourriture riche en fibres - Évitez le lait et les produits laitiers |
| Gr3 | Ik heb > 6 keer meer ontlasting dan normaal, de stoelgang is helemaal vloeibaar, zonder vaste stukjes OF de buikloop verstoort mijn dagelijks leven | Uw registratie is nu beschikbaar voor uw zorgteam. Indien u ongerust bent over dit symptoom, gelieve dan het centrum van uw behandelende arts te contacteren of bezoek de dienst spoedgevallen./ Votre enregistrement est maintenant disponible pour votre équipe de soins. Si ce symptôme vous inquiète, veuillez contacter le centre de votre médecin traitant ou vous rendre aux urgences. |

### Huiduitslag/ Réaction de peau

|  | **Graden** | **Verstrek info** |
| --- | --- | --- |
| Gr0 | Afwezig |  |
| Gr1 | Mijn huid ziet beperkt rood en/of ik heb milde plaatselijke jeuk/ Ma peau est un peu rouge et/ou elle me démange légèrement par endroits | - Neem geen lange, hete baden en gebruik vocht inbrengende   zeep en huid crème.   - Gebruik geen parfum, deodorant of aftershave met alcohol. - Bescherm uw huid en nagels door handschoenen te dragen bij huishoudelijk werk. - Vermijd de zon zo veel mogelijk, en gebruik anders een zonnecrème met een hoge beschermingsfactor en lippenbalsem. - Drink veel. - Draag katoenen kleren in plaats van wol of synthetisch materiaal. - Ne prenez pas de douches ou de bains brûlants et employez une crème - et un savon hydratants. - Bannissez le parfum, le déodorant ou l’after-shave à l’alcool. - Pour le ménage, protégez votre peau et vos ongles à l’aide de gants. - Restez le plus possible à l’écart du soleil. En cas d’exposition, utilisez une crème solaire à l’indice UV élevé et un baume à lèvres. - Buvez beaucoup. - Préférez les vêtements en coton à ceux en laine ou en synthétique. |
| Gr2 | Mijn huid ziet uitgebreid rood en/of ik heb af en toe last van ernstige jeuk/ Ma peau est rouge dans son ensemble et/ou elle me démange sérieusement de temps en temps | Idem als Gr 1 |
| Gr3 | Mijn huid ziet ernstig rood of er treedt vervelling op over (bijna) mijn hele lichaam en/of ik heb constant ernstige jeuk/ Ma peau est très rouge ou pèle sur (presque) tout mon corps et/ou je souffre constamment de démangeaisons sévères | Idem als Gr 1 |

### Huiduitslag (IT) / Réaction de peau (TI)

= Huiduitslag (non-IT) behalve Graad 3! => U zal de eerstvolgende werkdag worden gecontacteerd. Indien u sneller geholpen wenst te worden voor dit symptoom gelieve het centrum van uw behandelend arts te contacteren of bezoek de dienst spoedgevallen indien u ongerust bent. /Vous serez contacté(e) le premier jour ouvrable suivant. Souhaitez-vous recevoir de l’aide plus tôt pour ce symptôme ? Veuillez alors vous adresser au centre en relation avec votre médecin traitant ou rendez-vous aux urgences, si vous êtes inquiet(ète).

### Hoest/ Toux

|  | **Graden** | **Verstrek info** |
| --- | --- | --- |
| Gr0 | Geen hoest/ Pas de toux |  |
| Gr1 | Zeldzame droge hoest/ Toux sèche rare | Uw registratie werd opgenomen in uw oncologisch dossier. Bespreek dit symptoom bij een volgende consultatie of met uw oncologisch team. / Votre enregistrement a été repris dans votre dossier oncologique. Discutez de ce symptôme lors d’une prochaine consultation ou avec votre équipe oncologique. |
| Gr2 | Regelmatige droge hoest, gelijkaardig aan voorbije dagen/ Toux sèche régulière, comme celle des jours précédents | Idem Gr1 |
| Gr3 | Frequente droge hoest die stoort bij het slapen en dagelijkse activiteiten/ Toux sèche fréquente qui perturbe le sommeil et les activités quotidiennes | Idem Gr1 |

### Hoest (immunotherapie)/ Toux (immunothérapie)

= Hoest behalve Graad 3! => U zal de eerstvolgende werkdag worden gecontacteerd. Indien u sneller geholpen wenst te worden voor dit symptoom gelieve het centrum van uw behandelend arts te contacteren of bezoek de dienst spoedgevallen indien u ongerust bent. /Vous serez contacté(e) le premier jour ouvrable suivant. Souhaitez-vous recevoir de l’aide plus tôt pour ce symptôme ? Veuillez alors vous adresser au centre en relation avec votre médecin traitant ou rendez-vous aux urgences, si vous êtes inquiet(ète).

### Kortademigheid/Essouflement

Beg. tekst: Bent u de laatste dag(en) korter van adem?/ Avez-vous eu plus de problèmes d'essoufflement ces derniers jours ?

|  | **Graden** | **Verstrek info** |
| --- | --- | --- |
| Gr0 | Ik heb geen last van kortademigheid/ Je ne souffre pas d’essoufflement |  |
| Gr1 | Ik heb last van kortademigheid bij zware inspanningen/Je souffre d’essoufflement lors de gros efforts | - Vermijd erg zware inspanningen. - Indien deze klacht nieuw is, neemt u best snel contact op met uw huisarts of met uw behandelende arts. - Évitez les très gros efforts. - Si ce trouble est nouveau, il vaut mieux prendre rapidement contact avec votre généraliste ou votre médecin traitant. |
| Gr2 | Ik heb last van kortademigheid bij minimale inspanningen/ Je souffre d’essoufflement lors d’efforts minimes | - Vermijd de inspanningen die de klachten uitlokken. - Indien deze klacht nieuw is, neemt u best snel contact op met uw huisarts of met uw behandelende arts. - Évitez les très gros efforts. - Si ce trouble est nouveau, il vaut mieux prendre rapidement contact avec votre généraliste ou votre médecin traitant. |
| Gr3 | Ik ben kortademig in rust/ Je suis essoufflé au repos | - Neem onmiddellijk contact op met uw huisarts of behandelende arts in het ziekenhuis. - Indien uw arts telefonisch niet bereikbaar is, begeef u dan naar de dienst spoedgevallen. - Prenez immédiatement contact avec votre généraliste ou votre médecin traitant de l’hôpital. - Si votre médecin n’est pas joignable par téléphone, rendez-vous aux urgences. |

### Ontsteking in de mond/ Infection buccale

|  | **Graden** | **Verstrek info** |
| --- | --- | --- |
| Gr0 | Afwezig |  |
| Gr1 | Ik heb letsels of roodheid in de mond, zonder pijn OF ik heb pijn in de mond, maar ik heb geen letsels of roodheid/ Je souffre de lésions ou de rougeurs buccales indolores OU ma bouche me fait mal, mais je ne souffre d’aucune lésion ou rougeur | - Goede mondhygiëne:   - Raadpleeg een tandarts voor het starten met de therapie.   - Poets na elke maaltijd en voor het slapengaan uw tanden met een zachte tandenborstel.   - Verwijder uw kunstgebit bij pijn. Bewaar ‘s nachts uw kunstgebit droog in een daarvoor bestemd doosje.   - Tandenstokers niet gebruiken, eventueel tandflossdraadjes. - Hou uw mond vochtig:   - Drink regelmatig kleine hoeveelheden   - Zuig regelmatig op ijsblokjes   - Spoel regelmatig met zuiver water of mondspoeling op basis van chloorhexidine (bv. Perio-aid® 0,12% bij mucositus, Dentio 0,05% als onderhoudsdosis)   - Gebruik eventueel kunstspeeksel of spray van Vittel of Evian   - Gebruik eventueel suikervrije kauwgom   - Hou de lippen soepel door middel van een lippenstift - Voedingsadvies:   - Gebruik een gezonde, evenwichtige voeding   - Vermijd te warme, te gekruide, te zure spijzen en alcohol   - Vermijd harde en droge voeding   - Laat warme gerechten afkoelen   - Gebruik kleine maaltijden   - Drink water na alles wat u eet of drinkt   - Koude gerechten zoals roomijs worden als aangenaam ervaren   - Gebruik eventueel een rietje als u pijn hebt - Pour une bonne hygiène buccale :   - Consultez un dentiste avant de démarrer la thérapie.   - Après chaque repas et avant le coucher, brossez-vous les dents avec une brosse souple.   - Enlevez votre dentier si vous avez mal. La nuit, conservez-le au sec dans un étui destiné à cet effet.   - N’utilisez pas de cure-dents, éventuellement du fil dentaire. - Gardez votre bouche humide :   - Buvez régulièrement de petites quantités   - Sucez régulièrement des glaçons   - Rincez régulièrement à l’eau claire ou avec un bain de bouche à base de chlorhexidine (p.ex. Perio-aid® 0,12 % en cas de mucite, Dentio 0,05 % comme dose d’entretien)   - Utilisez éventuellement de la salive artificielle ou du spray Vittel ou Evian   - Mâchez éventuellement un chewing-gum sans sucre   - Maintenez vos lèvres souples au moyen d’un bâton à lèvres - Conseils nutritionnels :   - Optez pour une nourriture saine et équilibrée   - Évitez les aliments trop chauds, trop épicés, trop acides et l’alcool   - Évitez la nourriture dure et sèche   - Laissez refroidir les plats chauds   - Limitez les quantités   - Buvez de l’eau après tout ce que vous mangez ou buvez   - Les plats frois tels que les glaces sont appréciés   - Servez-vous éventuellement d’une paille si vous avez mal |
| Gr2 | Ik heb pijnlijke letsels, roodheid of zwelling in de mond, maar slikken is mogelijk/ Je souffre de lésions douloureuses, de rougeurs ou de gonflements buccaux, mais je peux avaler | Contacteer het ziekenhuis voor aanpassing van het mondspoelmiddel.   - Hou uw mond vochtig:   - Drink regelmatig kleine hoeveelheden.   - Zuig regelmatig op ijsblokjes.   - Spoel regelmatig met zuiver water of mondspoeling op basis van chloorhexidine (bv. Perio-aid®, Dentio 0,05%).   - Gebruik eventueel kunstspeeksel of spray van Vittel of Evian.   - Gebruik eventueel suikervrije kauwgom.   Contactez l’hôpital pour l’adaptation de votre produit de bain de bouche.   - Gardez votre bouche humide :   - Buvez régulièrement de petites quantités.   - Sucez régulièrement des glaçons.   - Rincez régulièrement à l’eau claire ou avec un bain de bouche à base de chlorhexidine (p.ex. Perio-aid®, Dentio 0,05 %).   - Utilisez éventuellement de la salive artificielle ou du spray Vittel ou Evian.   - Mâchez éventuellement un chewing-gum sans sucre. |
| Gr3 | Ik heb pijnlijke letsels, roodheid of zwelling in de mond, EN slikken, eten en/of drinken is moeilijk/onmogelijk/ Je souffre de lésions douloureuses, de rougeurs ou de gonflements buccaux, ET avaler, manger et/ou boire est difficile/impossible | Uw registratie is nu beschikbaar voor uw zorgteam. Indien u ongerust bent over dit symptoom, gelieve dan het centrum van uw behandelende arts te contacteren of bezoek de dienst spoedgevallen./ Votre enregistrement est maintenant disponible pour votre équipe de soins. Si ce symptôme vous inquiète, veuillez contacter le centre de votre médecin traitant ou vous rendre aux urgences. |

### Pijn/ Douleur

|  | **Graden** | **Verstrek info** |
| --- | --- | --- |
| Gr0 | Afwezig |  |
| Gr1 | Ik ervaar milde pijn, maar het verhindert mijn dagelijkse activiteiten niet/ Je souffre légèrement, mais cela n’empêche pas mes activités quotidiennes | Volg het advies van uw arts indien de pijn hinderlijk wordt. / Suivez les conseils de votre médecin si la douleur devient gênante. |
| Gr2 | Ik ervaar ondanks het nemen van de voorgeschreven pijnmedicatie matige pijn, die mijn dagdagelijkse activiteiten (zoals winkelen, koken) bemoeilijkt/ Malgré la prise des antidouleurs prescrits, je souffre modérément, ce qui gêne mes activités quotidiennes (telles que les courses, la cuisine) | Volg stipt het advies van uw arts met betrekking tot het nemen van de pijnmedicatie. Bespreek de ervaren pijn met uw arts tijdens volgend bezoek. Indien u sneller geholpen wenst te worden voor dit symptoom, gelieve het centrum van uw behandelend arts te contacteren./ Suivez scrupuleusement les conseils de votre médecin en ce qui concerne la prise des antidouleurs. Lors de la visite suivante, discutez de la douleur éprouvée avec votre médecin. Souhaitez-vous recevoir de l’aide plus tôt pour ce symptôme ? Veuillez vous adresser au centre en relation avec votre médecin traitant. |
| Gr3 | Ik ervaar ondanks het nemen van de voorgeschreven pijnmedicatie ernstige pijn, die mijn zelfzorg (zoals aankleden, wassen, eten) ernstig verstoort of verhindert/ Malgré la prise des antidouleurs prescrits, je souffre beaucoup, ce qui diminue gravement ou empêche mon autonomie (par exemple m’habiller, me laver, manger) | Blijf stipt het advies volgen van uw arts met betrekking tot het nemen van de pijnmedicatie. Uw registratie is nu beschikbaar voor uw zorgteam. Indien u ongerust bent over dit symptoom, gelieve dan het centrum van uw behandelende arts te contacteren of bezoek de dienst spoedgevallen./ Continuez à suivre scrupuleusement les conseils de votre médecin en ce qui concerne la prise des antidouleurs. Votre enregistrement est maintenant disponible pour votre équipe de soins. Si ce symptôme vous inquiète, veuillez contacter le centre de votre médecin traitant ou vous rendre aux urgences. |

### Regelmatige neusbloedingen/ Saignements de nez réguliers

|  | **Graden** | **Verstrek info** |
| --- | --- | --- |
| Gr0 | Afwezig |  |
| Gr1 | Minder dan 3x/week wat stipjes bloed op mijn zakdoek of sporadisch een neusbloeding/ Je trouve quelques taches de sang sur mon mouchoir moins de 3 x/semaine ou je saigne du nez sporadiquement | Ga rustig rechtop zitten met het hoofd licht naar voren gebogen en adem rustig door uw mond. Snuit de neus een keer goed uit. Knijp daarna de neus goed dicht gedurende tien minuten (let goed op de tijd). Plaats duim en wijsvinger aan weerszijden van de neus, vlak onder de plaats waar het bovenste harde stuk overgaat in het zachte gedeelte van de neus. Herhaal deze procedure een tweede keer, indien het niet voldoende effect geeft. / Asseyez-vous calmement et penchez légèrement la tête vers l’avant. Respirez tranquillement par la bouche et mouchez-vous bien une fois. Ensuite, pincez-vous le nez pendant dix minutes (faites bien attention à la durée). Placez le pouce et l’index des deux côtés du nez, juste sous l’endroit où la partie dure supérieure passe dans la partie molle. Si cette procédure ne donne pas d’effet suffisant, répétez-la. |
| Gr2 | 3-6x/week wat stipjes bloed op mijn zakdoek of sporadisch een neusbloeding/ Je trouve quelques taches de sang sur mon mouchoir 3-6 x/semaine ou je saigne du nez sporadiquement | Idem Gr1 |
| Gr3 | Het bloeden stopt niet na 2 keer 10 minuten de neus dichtgeknepen te hebben/ Le saignement ne s’arrête pas après avoir pincé le nez 2 fois 10 minutes | Uw registratie is nu beschikbaar voor uw zorgteam. Indien u ongerust bent over dit symptoom, gelieve dan het centrum van uw behandelende arts te contacteren of bezoek de dienst spoedgevallen./ Votre enregistrement est maintenant disponible pour votre équipe de soins. Si ce symptôme vous inquiète, veuillez contacter le centre de votre médecin traitant ou vous rendre aux urgences. |

### Slapeloosheid/ Insomnie

|  | **Graden** | **Verstrek info** |
| --- | --- | --- |
| Gr0 | Afwezig |  |
| Gr1 | Ik val moeilijk in slaap, maar slaap daarna wel redelijk/ Je m’endors difficilement, mais ensuite, je dors assez bien | Wanneer u langer dan ongeveer een half uur wakker ligt in bed, sta op en doe een rustgevende activiteit tot u weer slaperig wordt. Bij aanhoudend moeilijk inslapen of doorslapen, vraag advies bij de oncopsycholoog./ Lorsque vous restez éveillé au lit plus longtemps qu’une heure environ, levez-vous et livrez-vous à une activité apaisante jusqu’à ce que vous ayez de nouveau sommeil. En cas d’endormissement difficile ou de sommeil perturbé, consultez l’oncopsychologue. |
| Gr2 | Ik val moeilijk in slaap en word vaak wakker ’s nachts/ Je m’endors difficilement et me réveille souvent la nuit | Idem gr1 |
| Gr3 | Ik kan nog amper slapen/ Je peux à peine dormir | Idem gr1 |

### Spier- en/of gewrichtspijn/ Douleur musculaire / articulaire

|  | **Graden** | **Verstrek info** |
| --- | --- | --- |
| Gr0 | Afwezig |  |
| Gr1 | Milde pijn, maar het verhindert mijn dagelijkse activiteiten niet/ Douleur légère, mais qui n’empêche pas mes activités quotidiennes | Neem eventueel een pijnstiller op basis van paracetamol (max 4 x 1 g per dag) en beweeg voldoende./ Prenez éventuellement un antidouleur à base de paracétamol (max. 4 x 1 g par jour) et bougez-vous suffisamment. |
| Gr2 | Matige pijn, dagelijkse activiteiten lukken nog, maar ik functioneer moeilijker/ Douleur modérée qui n’empêche pas mes activités quotidiennes, mais rend mon fonctionnement difficile | Neem eventueel een pijnstiller op basis van paracetamol (max 4 x 1 g per dag) en beweeg voldoende. Bespreek dit symptoom met uw behandelende arts./ Prenez éventuellement un antidouleur à base de paracétamol (max. 4 x 1 g par jour) et bougez-vous suffisamment. Discutez de ce symptôme avec votre médecin traitant. |
| Gr3 | Ernstige pijn, uitvoeren van dagelijkse activiteiten lukt nauwelijks of niet meer/ Douleur sévère qui rend mes activités quotidiennes très difficiles ou impossibles | Uw registratie is nu beschikbaar voor uw zorgteam. Indien u ongerust bent over dit symptoom, gelieve dan het centrum van uw behandelende arts te contacteren of bezoek de dienst spoedgevallen./ Votre enregistrement est maintenant disponible pour votre équipe de soins. Si ce symptôme vous inquiète, veuillez contacter le centre de votre médecin traitant ou vous rendre aux urgences. |

### Tintelingen vingers/tenen/ Picotements main/pied

|  | **Graden** | **Verstrek info** |
| --- | --- | --- |
| Gr0 | Geen |  |
| Gr1 | Ik heb hier last van, maar het verstoort mijn functioneren niet/ Je souffre de picotements ou fourmillements mais cela ne perturbe pas mes activités quotidiennes | Wie negatief reageert op koude prikkels zoekt natuurlijk het best de warmte op. Kleed je goed aan en draag sokken of handschoenen. / Nous recommandons au patient qui réagit négativement aux stimuli froids de rechercher la chaleur. Habillez-vous bien et portez des chaussettes ou des gants. |
| Gr2 | Ik heb hier last van en sommige van mijn dagelijkse activiteiten (bv. koken, winkelen) lukken daardoor moeilijker/ Je souffre des picotements ou fourmillements et certaines de mes activités quotidiennes (cuisine, courses, etc.) sont plus difficiles à faire à cause de cela | Idem Gr 1 |
| Gr3 | Ik heb hier last van en het verstoort mijn zelfzorg (bv. wassen, aankleden)/ Je souffre des picotements ou fourmillements et perturbe mes soins personelles (ex. me laver, m’habiller) | Uw registratie is nu beschikbaar voor uw zorgteam. Indien u ongerust bent over dit symptoom, gelieve dan het centrum van uw behandelende arts te contacteren of bezoek de dienst spoedgevallen./ Votre enregistrement est maintenant disponible pour votre équipe de soins. Si ce symptôme vous inquiète, veuillez contacter le centre de votre médecin traitant ou vous rendre aux urgences. |

### Verminderde eetlust/ Perte d’appétit

|  | **Graden** | **Verstrek info** |
| --- | --- | --- |
| Gr0 | Afwezig |  |
| Gr1 | Ik ben misselijk en/of ik heb minder eetlust, maar ik hoef mijn eetgewoonten niet aan te passen/ J’ai la nausée et/ou j’ai moins d’appétit, mais ne dois pas modifier mes habitudes alimentaires | - Zorg voor een aangename en rustige eetomgeving. - Neem geen lightproducten of voedingsmiddelen met minder suikers en/of vetten, kies voor de volwaardige alternatieven. - Eetlustopwekkende voedingsmiddelen zijn fruitsap en aperitief (in beperkte mate), bouillon, sorbet, schijfjes appel, kauwgom,… - Breng veel variatie in je voeding. - Kauw goed en vermijd het inslikken van lucht. - Vermijd grote hoeveelheden vezelrijke voeding, wegens het snel optredend verzadigingsgevoel. - Hou individueel verpakte en favoriete snacks binnen handbereik. - Drink niet vlak vóór of tijdens de maaltijd. - Neem soep niet bij, maar een uur voor de maaltijd. - Drink voldoende, bij voorkeur water. - Mangez dans un environnement agréable et calme. - Préférez les aliments complets aux produits allégés ou aux aliments contenant moins de sucre et/ou de graisses. - Des aliments appétissant sont : les jus de fruits et les apéritifs (avec modération), le bouillon, les sorbets, les tranches de pommes, le chewing-gum... - Variez beaucoup votre alimentation. - Mâchez bien et n’avalez pas d’air. - Évitez les grandes quantités d’aliments riches en fibres à cause de la sensation de satiété rapide. - Gardez toujours vos en-cas favoris emballés individuellement à portée de main. - Ne buvez pas juste avant le repas ou pendant celui-ci. - Ne consommez pas de soupe avec le repas, mais bien une heure avant. - Buvez suffisamment, de préférence de l’eau. |
| Gr2 | Ik eet en/of drink minder, maar ik verlies geen gewicht/ Je mange et/ou je bois moins, mais je ne perds pas de poids | Idem Gr1 |
| Gr3 | Ik kan niet meer voldoende eten en/of drinken, ik verlies gewicht/ Je ne peux plus manger et/ou boire assez ; je perds du poids | Idem Gr1 |

### Vermoeidheid/ Fatigue

|  | **Graden** | **Verstrek info** |
| --- | --- | --- |
| Gr0 | Ik ben niet meer vermoeid dan normaal/ Je ne suis pas plus fatigué(e) que la normale |  |
| Gr1 | Ik ben licht vermoeid of futloos, maar het hindert mijn dagelijkse activiteiten niet/ Je suis légèrement fatigué(e) ou sans énergie, mais cela n’empêche pas mes activités quotidiennes | - Probeer een evenwicht te vinden tussen (lichte) activiteit en rust. - Regelmatig lichte oefeningen zijn beter dan erg belastende activiteiten. - Plan uw activiteiten en taken, stel prioriteiten. - Las tijdens de dag voldoende rustperiodes in (max. 20 tot 30 minuten), liefst in de zetel. - Vraag hulp aan familie, vrienden of thuiszorgwerkers. Verdeel de taken. - Lees de folder “vermoeidheid bij en na kanker”, hier kan je praktische tips terugvinden. - Volg een infosessie rond “omgaan met vermoeidheid” in het ziekenhuis. - Essayez de trouver un équilibre entre activité (légère) et repos. - Des activités légères régulières valent mieux que des activités très lourdes. - Planifiez vos activités et tâches, fixez des priorités. - Offrez-vous suffisamment de périodes de repos pendant la journée (max. 20 à 30 minutes), de préférence dans un fauteuil. - Demandez de l’aide à votre famille, amis ou travailleurs sociaux à domicile. Répartissez les tâches. - La brochure consacrée à « La fatigue liée au cancer et à la période suivant ce dernier » vous fournira des conseils pratiques. - Suivez une séance d’information au sujet de « La gestion de la fatigue » à l’hôpital. |
| Gr2 | Ik ben matig vermoeid of futloos, uitvoeren van dagelijkse activiteiten (bv. koken, winkelen) gaan moeilijker/ Je suis modérément fatigué(e) ou paresseu(x)(sse) cela perturbe mes tâches quotidiennes (ex.: cuisiner, faire les courses) | Idem als Gr1 |
| Gr3 | Ik ben zo vermoeid of futloos dat het uitvoeren van mijn zelfzorg (bv. wassen, aankleden) nauwelijks of niet meer lukt/ Je suis si fatiguée ou paresseu(x)(sse) ceci m’empêche de prendre soin de moi (ex. : me laver, manger) | Idem als Gr1 |

### Constipatie/ Constipation

|  | **Graden** | **Verstrek info** |
| --- | --- | --- |
| Gr0 | Afwezig |  |
| Gr1 | Ik kan enkel ontlasting maken als ik af en toe een hulpmiddel gebruik (laxeermiddel, fruit, …)/ Seul un remède (laxatif, fruit…) pris de temps en temps me permet d’aller à selles | - Drink 1,5 tot zelfs 2 liter per dag, bij voorkeur water, anders heeft een extra vezelinname geen zin en kan de constipatie zelfs nog toenemen. - Neem kleine, frequente maaltijden - Neem voldoende tijd om naar het toilet te gaan - Beweeg voldoende indien mogelijk, bijvoorbeeld door te wandelen en te fietsen. Bent u bedlegerig, dan kunnen oefeningen te bed helpen, zoals bewegen van armen en benen en regelmatig van houding veranderen. - Drink vóór het ontbijt een glas lauw water of vers vruchtensap. - Eet op geregelde tijdstippen en sla vooral het ontbijt niet over. - Dadels, abrikozen en pruimen stimuleren de darmen. - Kies voedingsmiddelen rijk aan voedingsvezels zoals bruin en volkorenbrood, volkorenbeschuit, volkorenkoekje, graanproducten zoals havermout, muesli, drinkontbijt met voedingsvezel, peulvruchtensoepen (erwtensoep, bruine bonensoep), vers fruit, vruchtenmoes, vruchtendrank met extra voedingsvezel, groenten, zowel gekookt als rauw, aardappelen, (volkoren)pasta, (zilvervlies) rijst. - Voeg extra zemelen of voedingsvezelsupplementen (verkrijgbaar bij de apotheek of in de natuurwinkel) toe aan granen, fruitsap, melk, yoghurt en soepen. - Eet geen chocolade, kaas en eieren. - Buvez 1,5 voire 2 litres par jour, de préférence de l’eau. Sinon, une consommation supplémentaire de fibres n’a aucun sens et peut même augmenter la constipation. - Mangez fréquemment et en petites quantités - Prenez assez de temps pour aller aux toilettes - Si possible, bougez suffisamment, par exemple en vous promenant ou en faisant du vélo. Si vous êtes alité(e), faites des exercices tels que bouger les bras et les jambes et changer régulièrement de position. - Avant le petit-déjeuner, buvez un verre d’eau tiède ou de jus de fruits frais. - Mangez à des heures régulière et ne passez surtout pas le petit-déjeuner. - Les dates, les abricots et les prunes stimulent les intestins. - Choisissez des aliments riches en fibres tels que le pain gris et le pain complet, les biscottes et les biscuits complets, les céréales telles que le gruau d’avoine, le muesli, le petit-déjeuner à boire contenant des fibres, les soupes aux légumes (soupe aux pois, soupe aux haricots bruns), les fruits frais, les compotes, les boissons aux fruits très riches en fibres, les légumes cuits ou crus, les pommes de terre, les pâtes (complètes), le riz (complet). - Ajoutez du son supplémentaire ou des compléments alimentaires (disponibles en pharmacie où au magasin d’aliments naturels) aux céréales, aux jus de fruits, au lait, aux yogourts et aux soupes. - Bannissez le chocolat, le fromage et les œufs. |
| Gr2 | Ik kan enkel ontlasting maken als ik dagelijks laxeermiddelen gebruik/ Seul un laxatif pris tous les jours me permet d’aller à selles | Idem Gr 1 |
| Gr3 | De verstopping verstoort mijn dagelijkse activiteiten en laxeermiddelen helpen niet/ La constipation perturbe mes activités quotidiennes et les laxatifs ne m’aident pas | Uw registratie is nu beschikbaar voor uw zorgteam. Indien u ongerust bent over dit symptoom, gelieve dan het centrum van uw behandelende arts te contacteren of bezoek de dienst spoedgevallen./ Votre enregistrement est maintenant disponible pour votre équipe de soins. Si ce symptôme vous inquiète, veuillez contacter le centre de votre médecin traitant ou vous rendre aux urgences. |

### Gehoor/ Audition

|  | **Graden** | **Verstrek info** |
| --- | --- | --- |
| Gr0 | Ik heb geen problemen met mijn gehoor/ Mon audition ne me pose aucun problème |  |
| Gr1 | Ik heb last van oorsuizen zonder impact op mijn dagelijks functioneren en/of het gevoel dat ik minder hoor zonder dat het impact heeft op mijn dagelijks functioneren/ Je souffre d’acouphènes qui n’affectent pas mon fonctionnement quotidien et/ou j’éprouve la sensation que j’entends moins, sans que cela influence mon fonctionnement quotidien | Uw registratie werd opgenomen in uw oncologisch dossier. Bespreek dit symptoom bij een volgende consultatie of met uw oncologisch team./ Votre enregistrement a été repris dans votre dossier oncologique. Discutez de ce symptôme lors d’une prochaine consultation ou avec votre équipe oncologique. |
| Gr2 | Ik heb last van oorsuizen met impact op mijn dagelijks functioneren en/of het gevoel dat ik minder hoor met impact op mijn dagelijks functioneren/ Je souffre d’acouphènes qui affectent mon fonctionnement quotidien et/ou j’éprouve la sensation que j’entends moins, ce qui influence mon fonctionnement quotidien | Idem Gr 1 |
| Gr3 | Ik heb last van oorsuizen met zeer uitgesproken impact op mijn dagelijks functioneren en of het gevoel dat ik minder hoor met uitgesproken impact op mijn dagelijks functioneren/ Je souffre d’acouphènes qui affectent énormément mon fonctionnement quotidien et/ou j’éprouve la sensation que j’entends moins, ce qui influence fortement mon fonctionnement quotidien | Idem Gr 1 |

### Psychologische/emotionele belasting/ Charge psychologique/émotionnelle

|  | **Graden** | **Verstrek info** |
| --- | --- | --- |
| Gr0 | Ik voel me goed, ik denk (bijna) niet aan mijn ziekte of behandeling/ Je me sens bien, je ne pense (presque) pas à ma maladie ou à mon traitement |  |
| Gr1 | Ik voel me minder goed, ik pieker meer, ik huil af en toe, ik ben iets prikkelbaarder of angstiger, maar het verstoort mijn dagelijks leven niet/ Je me sens moins bien, je rumine davantage, je pleure de temps en temps, je suis plus irritable ou anxieux(se), mais cela ne perturbe pas ma vie quotidienne | Meld dit bij uw volgend bezoek zeker aan uw oncocoach om samen te bekijken of een consultatie bij een oncopsycholoog wenselijk is./ Ne manquez pas de le mentionner à votre oncocoach afin d’examiner ensemble l’opportunité d’une consultation chez un oncopsychologue. |
| Gr2 | Ik voel me niet goed, ik pieker veel, huil vaak, ik ben prikkelbaarder of angstiger, het verstoort me tijdens mijn dagelijkse activiteiten, ontspanning of slaap/ Je ne me sens pas bien et j’ai le sentiment de ne pouvoir faire face : je rumine et pleure constamment, je suis très irritable et anxieux(e) ; je ne peux donc plus me livrer à mes activités quotidiennes, ma détente ou mon sommeil | Idem Gr1 |
| Gr3 | Ik voel me niet goed en ik heb het gevoel het niet aan te kunnen: ik pieker en huil constant, ik ben erg prikkelbaar en erg angstig en ik kom hierdoor niet meer toe aan mijn dagelijkse activiteiten, ontspanning of slaap/Je ne me sens pas bien et j’ai le sentiment de ne pouvoir faire face : je rumine et pleure constamment, je suis très irritable et anxieux(e) ; je ne peux donc plus me livrer à mes activités quotidiennes, ma détente ou mon sommeil | Idem Gr1 |

### Haarverlies/ Perte de cheveux

|  | **Graden** | **Verstrek info** |
| --- | --- | --- |
| Gr0 | Geen haarverlies/ Aucune perte de cheveux |  |
| Gr1 | Ik heb last van iets dunner haar | - Gebruik een grove kam of zachte borstel en probeer het gebruik van föhn of krultang te beperken. - Het gebruik van een conditioner kan helpen haarverlies tijdens kammen of borstelen tegen te gaan. - Was je haar met lauw water, dep je haar droog. - Vermijd chemische stylingproducten en haarverven. - Vermijd stress. - Eet vers fruit, verse groenten, eiwitrijke voeding (vlees, vis, eieren, peulvruchten) en omega 3-vetzuren (zalm, makreel, haring). - Beperk het gebruik van cafeïne. - Probeer te stoppen met roken. - Servez-vous d’un peigne à dents larges ou d’une brosse douce et essayez de limiter l’usage d’un sèche-cheveux ou d’un fer à boucler. - Un après-shampooing peut contribuer à empêcher la chute de cheveux lors du peignage ou du brossage. - Lavez-vous les cheveux à l’eau tiède et séchez-les en les épongeant. - Bannissez les produits chimiques de coiffage et les colorations. - Évitez le stress. - Mangez des fruits et des légumes frais, des aliments riches en protéines (viande, poisson, œufs, légumes) et en acides gras oméga 3 (saumon, maquereau, hareng). - Réduisez votre consommation de caféine. - Essayez d’arrêter de fumer. |
| Gr2 | Ik heb minder dan 50% van mijn haren verloren/ Perte de ± 50 % des cheveux | - Gebruik een grove kam of zachte borstel en probeer het gebruik van föhn of krultang te beperken. - Het gebruik van een conditioner kan helpen haarverlies tijdens kammen of borstelen tegen te gaan. - Was je haar met lauw water, dep je haar droog. - Vermijd chemische stylingproducten en haarverven. - Vermijd stress. - Eet vers fruit, verse groenten, eiwitrijke voeding (vlees, vis, eieren, peulvruchten) en omega 3-vetzuren (zalm, makreel, haring). - Beperk het gebruik van cafeïne. - Probeer te stoppen met roken. - Spreek erover met uw arts, eventueel wordt u verwezen naar een dermatoloog. - Servez-vous d’un peigne à dents larges ou d’une brosse douce et essayez de limiter l’usage d’un sèche-cheveux ou d’un fer à boucler. - Un après-shampooing peut contribuer à empêcher la chute de cheveux lors du peignage ou du brossage. - Lavez-vous les cheveux à l’eau tiède et séchez-les en les épongeant. - Bannissez les produits chimiques de coiffage et les colorations. - Évitez le stress. - Mangez des fruits et des légumes frais, des aliments riches en protéines (viande, poisson, œufs, légumes) et en acides gras oméga 3 (saumon, maquereau, hareng). - Réduisez votre consommation de caféine. - Essayez d’arrêter de fumer. - Parlez-en à votre médecin qui vous renverra le cas échéant à un dermatologue. |
| Gr3 | Ik heb meer dan 50% van mijn haren verloren / Perte de plus de 50 % des cheveux | Idem Gr2 |

Supplemental Table 2:

Clinical features of patients in both the AMTRA and BAPIC system during SARS-Cov-2 pandemic and lockdown.

|  |  | AMTRA (n=79) | BAPIC (n= 281) |
| --- | --- | --- | --- |
| SEX | |  |  |
|  | Men | 48 (60.8%) | 156 (55.5%) |
|  | Women | 31 (39.2%) | 125 (44.5%) |
| AGE in years median (range) | | 63 (26-83) | 62 (21-91) |
| TUMOR TYPE | |  |  |
|  | Breast | 2 (2.5%) | 56 (19.9%) |
|  | Central Nervous System | 2 (2.5%) | 3 (1.1%) |
|  | Colorectal Cancer | 24 (30.3%) | 25 (8.9%) |
|  | Head and Neck Cancer | 10 (12.7%) | 17 (6.0%) |
|  | Lung Cancer | 13 (16.5%) | 57 (20.3%) |
|  | Thymoma | 0 (0%) | 2 (0.7%) |
|  | Gastric Cancer | 1 (1.3%) | 0 (0%) |
|  | Esophageal/ GUS Cancer | 3 (3.8%) | 3 (1.1%) |
|  | Liver/ Cholangio Carcinoma | 8 (10.1%) | 6 (2.1%) |
|  | Pancreatic Cancer | 10 (12.7%) | 8 (2.8%) |
|  | Melanoma | 1 (1.3%)) | 11 (3.9%) |
|  | Bladder Cancer | 0 (0%) | 12 (4.3%) |
|  | Renal Cell Cancer | 1 (1.3%) | 4 (1.4%) |
|  | Penile Cancer | 0 (0%) | 1 (0.4%) |
|  | Prostate Cancer | 3 (3.8%) | 9 (3.2%) |
|  | Haematology | 0 (0%) | 23 (8.2%) |
|  | Gynaecological | 0 (0%) | 32 (11.4%) |
|  | Soft Tissue and Bone Sarcoma | 0 (0%) | 7 (2.5%) |
|  | Not Specified | 1 (1.3%) | 5 (1.8%) |
| THERAPEUTIC INTENT | |  |  |
|  | Adjuvant | 3 (3.8%) | NA |
|  | Metastatic /Locally advanced | 44 (55.7%) | NA |
|  | Unknown | 32 (40.5%) | NA |

Supplemental Figure 1
